# Neurological Recovery After Complete Paraplegia Due to Metastatic Prostate Cancer: A Case Report

**DOI:** 10.7759/cureus.108509

**Published:** 2026-05-08

**Authors:** Prabhat Poudel, Filipe PiazziTavares, Zachary L Tataryn, Serdar Kaya, Ekkehard M Kasper

**Affiliations:** 1 Neurological Surgery, Boston Medical Center, Boston, USA; 2 Neurosurgery, Boston Medical Center Brighton, Boston, USA; 3 Faculty of Health Sciences, McMaster University, Hamilton, Ontario, CAN

**Keywords:** case report, epidural hematoma, laminectomy, metastatic epidural spinal cord compression, neurological recovery, paraplegia, prostate cancer, spinal fusion

## Abstract

Loss of motor function due to metastatic epidural spinal cord compression (MESCC) is a time-sensitive oncologic emergency. Rapid recognition and decompression are essential, yet complete paraplegia often portends poor recovery. We report a case of severe MESCC secondary to metastatic prostate adenocarcinoma, complicated by a postoperative epidural hematoma, which achieved near-complete recovery following timely surgical intervention and multidisciplinary management. The patient was a 70-year-old gentleman with newly diagnosed metastatic prostate cancer in the upper thoracic spine, who presented with rapidly progressing bilateral leg weakness evolving to complete paraplegia within 3 days, and displayed a T4 sensory level and incontinence. He underwent emergent laminectomy, decompression, tumor excision, and instrumented fusion. He began regaining voluntary movement by postoperative day one, which further improved over the next few days. On day three, neurological decline prompted a repeat MRI, revealing a postoperative thoracic epidural hematoma, which required evacuation. Following surgery, the patient exhibited good neurological recovery. He was discharged to acute rehab on androgen deprivation therapy. After six weeks, he had regained full strength. This case highlights that meaningful recovery can occur from complete paraplegia secondary to MESCC, provided that timely decompression, stabilization, hemodynamic optimization, and vigilant postoperative monitoring are ensured.

## Introduction

Metastatic epidural spinal cord compression (MESCC) occurs in approximately 5-7% of cancer patients and represents a neurosurgical emergency requiring urgent decompression, often in combination with instrumented stabilization to preserve neurological function [1,2]. Prostate cancer is the most common solid organ malignancy in men and is a leading cause of spinal metastases, most frequently involving the thoracic spine [3]. Once paraplegia develops, the prognosis for recovery is generally considered poor. However, timely intervention can occasionally reverse complete deficits [4,5].

Postoperative epidural hematoma is an uncommon complication of spinal surgery but can rapidly cause myelopathy by itself and irreversibly injure the spinal cord if not promptly addressed [6,7]. Hereby, we present a case of metastatic prostate adenocarcinoma causing complete paraplegia as well as sensory and autonomous myelopathy due to thoracic MESCC. The index surgery, however, was complicated by postoperative epidural hematoma, which was managed successfully with emergent intervention for evacuation, resulting in excellent neurological recovery.

## Case presentation

**Figure 1:**
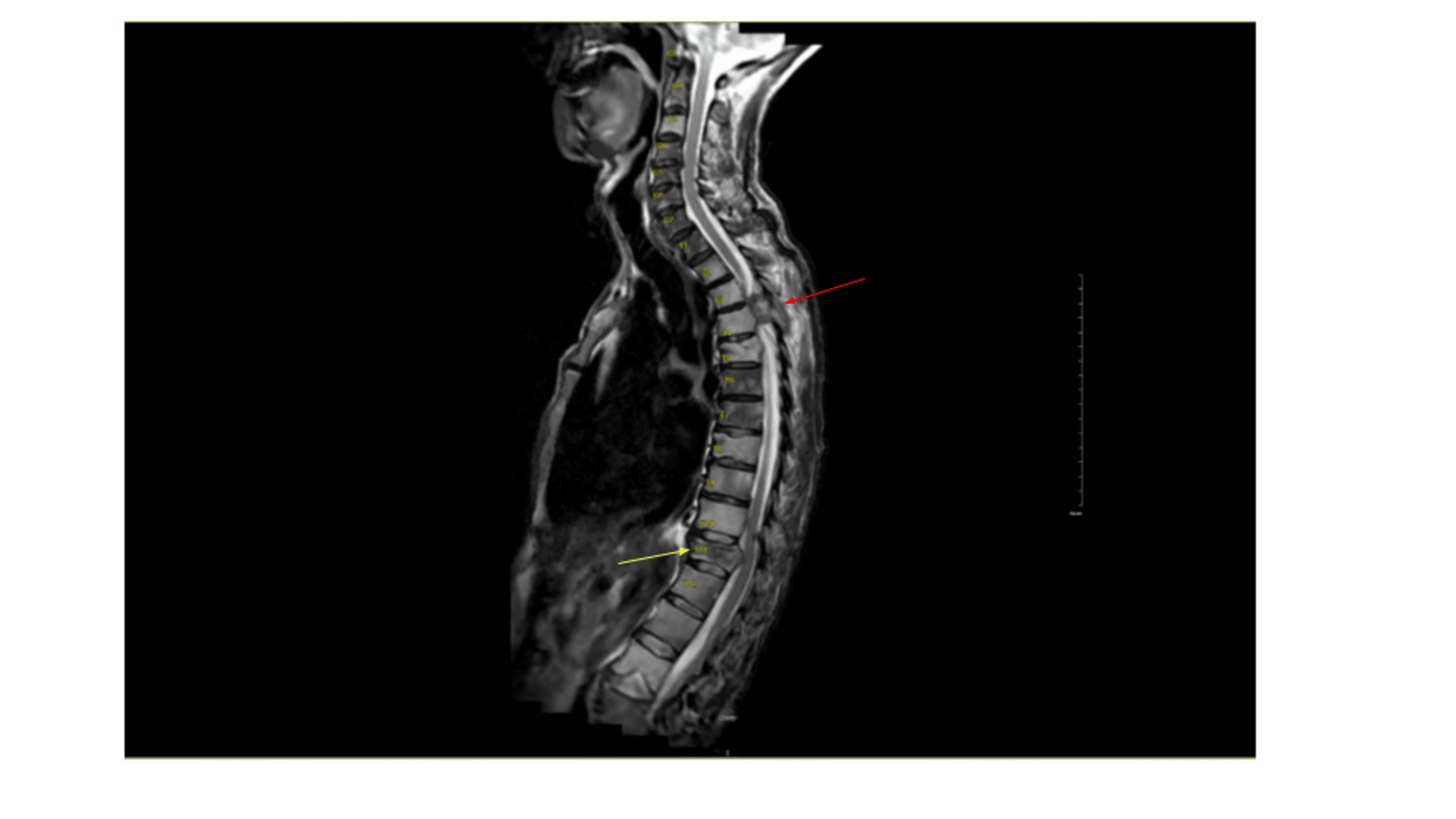
MRI of the spine demonstrating metastatic disease from T3 to T5 (red arrow) causing severe cord compression and a pathologic T11 fracture (yellow arrow)

A 70-year-old man with metastatic prostate adenocarcinoma involving multiple sites, including the spine, pelvis, femurs, and ribs, presented to our emergency department with rapidly progressive bilateral leg weakness and incontinence. At presentation, he had not yet initiated systemic therapy or radiation; androgen-deprivation therapy with bicalutamide and leuprolide was initiated during the postoperative period. His symptoms began with right foot weakness three days prior to admission, rapidly progressing to complete bilateral lower-extremity paralysis with numbness below the nipple line and new bowel and bladder incontinence. On initial examination, upper extremity strength was full in all muscle groups (5/5), whereas lower extremity muscles had no contractions or movements (0/5). Bilateral reflexes were brisk, with complete loss of sensation below the nipple line, including the genital and perianal region, and absent rectal tone, consistent with an American Spinal Injury Association (ASIA) A complete injury. MRI of the spine was remarkable for expansile metastatic disease from T3 to T5, causing severe cord compression, a pathologic T11 fracture with moderate ventral compression (Figure 1). CT scan confirmed extensive osseous and peritoneal metastases. PSA was 831 ng/mL. The patient had not yet initiated systemic therapy. 

**Figure 2:**
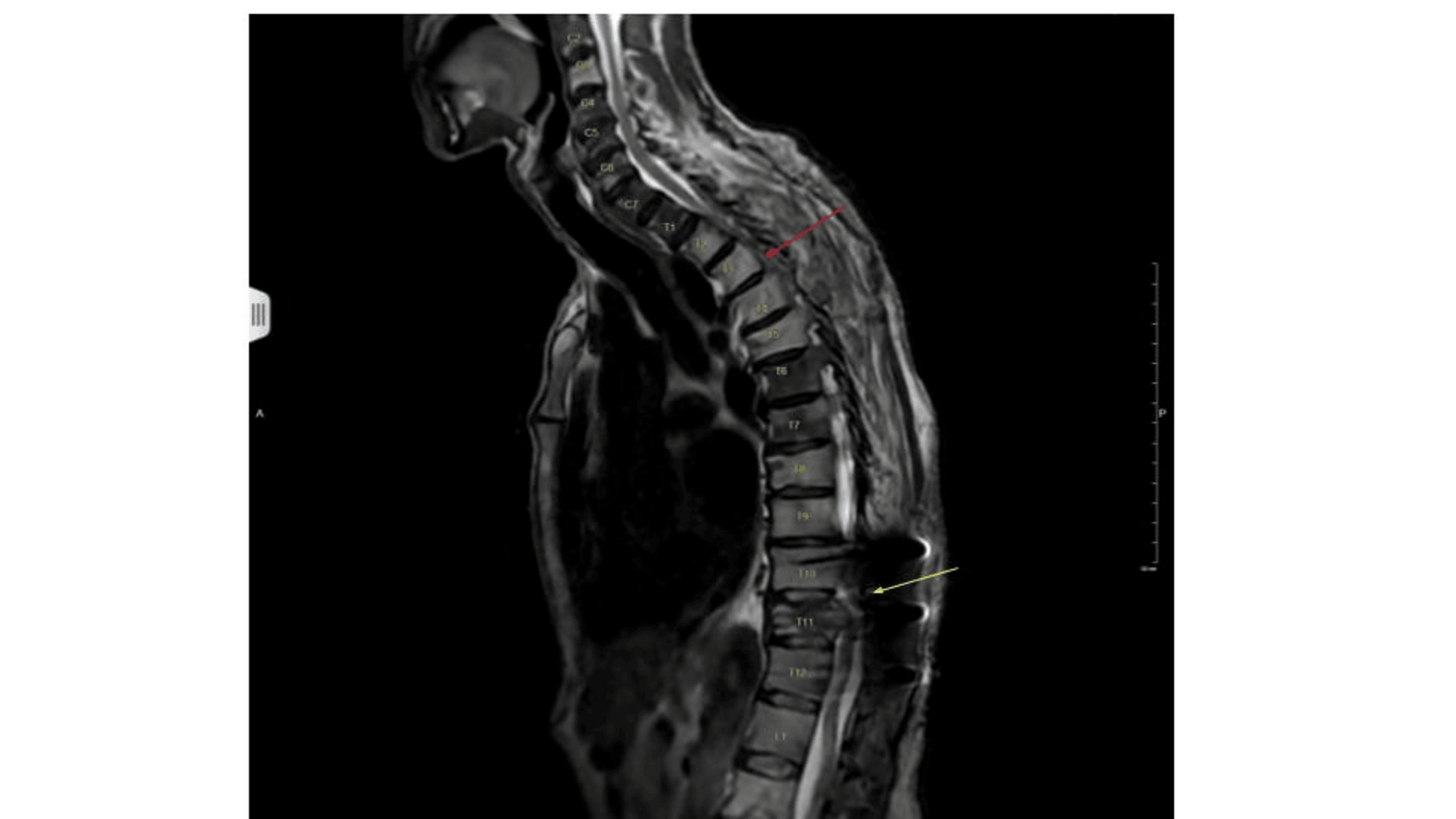
Postoperative MRI of the spine after T2-T4 laminectomy (red arrow) and T10-T11 laminectomy with tumor debulking and T10-T12 posterior instrumented fusion (yellow arrow)

He underwent simultaneous decompressions at T2 to T4 and T10 to T11 via wide laminectomies for tumor debulking and subsequent T10 to T12 posterior instrumented fusion (Figure 2).

The tumor was highly vascular in nature, resulting in significant intraoperative blood loss of approximately 1600 ml, requiring intraoperative transfusion of 5 units of packed red blood cells, as well as platelets and cryoprecipitate. After meticulous hemostasis, two subfascial drains were placed prior to closure. No intraoperative neurophysiologic signals were observed in the lower extremities. Immediately after the surgery, he was admitted to the surgical intensive care unit (SICU) and managed with a mean arterial pressure (MAP) target ≥75 mmHg using norepinephrine and vasopressin to improve spinal cord perfusion and dexamethasone to reduce cord edema. Neurologically, he remained ASIA A immediately postoperatively but demonstrated trace voluntary hip and knee movements within 24 hours. During this period, the patient required additional postoperative transfusions for anemia. Drain output progressively diminished, and both subfascial drains were removed on postoperative day 2. Over the subsequent postoperative days, neurological function improved further, with the patient demonstrating progressive proximal lower-extremity movement, occasional toe wiggling, and the ability to move both legs while positioned on the bed, although he remained unable to lift them against gravity.

On postoperative day 3, his neurological exam worsened again. MRI revealed an extended posterior thoracic epidural hematoma with resulting cord compression. Urgent evacuation of the hematoma was performed. A thick clot that was putting pressure on the spinal cord was identified and successfully removed, hemostasis was achieved, and new wound drains were placed. Following re-exploration and hematoma evacuation, neurological improvement resumed. Over the next three days, lower-extremity strength improved from trace to antigravity (4/5), and by six weeks, he regained near-full motor function. Sensation also normalized. However, intermittent catheterization remained necessary for neurogenic bladder management.

**Figure 3:**
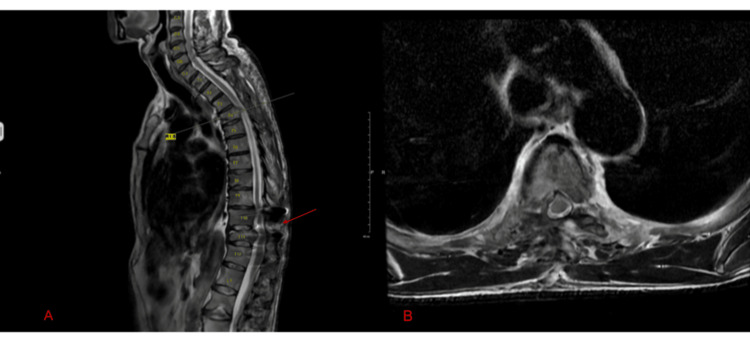
A: Thoracic MRI saggital view demonstrating adequate decompression with minor signal changes at T10-T11 levels (red arrow); B: Axial view through T4 demonstrating adequate decompression

The patient was then started on bicalutamide and leuprolide. Multimodal analgesia and physical therapy were initiated, and after significant in-hospital recovery, he was discharged for short-term rehabilitation. At his six-week follow-up visit, a thoracic MRI demonstrated successful decompression but evidence of a postoperative signal change indicative of myelomalacia at T10 to T11 (Figure 3).

Conventional radiographs showed adequate instrumentation. As of now, the patient continues androgen deprivation therapy and rehabilitation. At the most recent six-week follow-up visit, neurological examination demonstrated full strength in both upper extremities (5/5). In the lower extremities, iliopsoas strength was 4+/5, quadriceps 5-/5, and distal muscle groups 5/5, with intact sensation.

## Discussion

MESCC from prostate cancer represents a major cause of neurological disability in patients with advanced malignancy. Timely diagnosis and intervention are essential to preserve function and quality of life. Complete paraplegia has historically portended poor outcomes. Patchell et al. demonstrated improved outcomes when surgical decompression plus radiotherapy was pursued in comparison to radiotherapy alone [8].

After ASIA A injuries, patients undergoing early surgery can have a higher rate of improvement (46.1%) than patients undergoing late surgery (25%), but complete recovery remains very rare [5,9]. Our case adds to the limited literature documenting substantial neurological recovery despite initial complete paraplegia, emphasizing that aggressive decompression and postoperative optimization can yield meaningful improvement.

Neurocritical care management postoperatively is considered essential, and maintenance of MAP ≥75-85 mmHg is associated with improved spinal cord perfusion and neurological outcomes in acute spinal cord injury [10]. This patient’s strict perfusion protocol and vasopressor support for three days after the surgery may have contributed to the favorable recovery trajectory.

This patient’s dire situation was further complicated by the development of a postoperative spinal epidural hematoma, which is a rare but well-described and often devastating complication after spinal surgery. Postoperative spinal epidural hematoma is a rare but well-described complication after spinal surgery, with several recognized perioperative risk factors [6,7]. For that very reason, we implanted subfascial wound drains at the time of the index surgery. However, these drains were removed after 48 hrs. Early recognition and prompt evacuation of a spinal epidural hematoma (EDH) are critical; delays can result in permanent deficits [11]. In this case, early re-imaging and emergent re-exploration allowed for adequate repeat decompression, which facilitated further neurological improvement.

With the report on the successful management of this patient, we demonstrate the neurological reversibility of complete paralysis secondary to MESCC if managed with prompt and aggressive treatment. This case also illustrates the need for ongoing vigilance in postoperative monitoring during the recovery phase, since early detection and management of the evolving epidural hematoma were essential for the positive outcome.

## Conclusions

In metastatic prostate cancer-related MESCC, complete paraplegia does not preclude meaningful recovery when timely decompression and stabilization are provided. Furthermore, we want to emphasize that postoperative vigilance is essential, since complications such as EDH may occur, and the need for additional interventions can arise, requiring prompt attention and execution. Postoperative epidural hematoma must be suspected in patients with newly emerging neurological deficits. This case underscores the importance of early surgery, postoperative care optimization via MAP augmentation, and meticulous follow-up to optimize chances for a good neurological outcome.
